# Development of a Generic Bio-Interface for Immuno-Biodetection on an Oxide Surface Targeting Pathogen Bacteria

**DOI:** 10.3390/molecules30183681

**Published:** 2025-09-10

**Authors:** Thibaut Zwingelstein, Thérèse Leblois, Vincent Humblot

**Affiliations:** Université Marie et Louis Pasteur, CNRS Institut FEMTO-ST, CNRS, MicroNano Sciences and Systems Department (MN2S), 25000 Besançon, France; thibaut.zwingelstein@femto-st.fr (T.Z.); therese.leblois@femto-st.fr (T.L.)

**Keywords:** biointerface, lithium niobate, specific bacteria detection, pathogen bacteria biosensor

## Abstract

With the increase in contamination by microbial agents (bacteria, viruses, etc.) in the fields of agri-food, healthcare, and environment, it is necessary to detect and quantify these biological elements present in complex fluids in a short time with high selectivity, high sensitivity, and, if possible, moderate cost. Acoustic wave biosensors, based on immuno-detection, appear to meet a certain number of these criteria. In this context, we are developing a generic antibody-based biointerface that can detect a wide range of pathogenic bacterial agents using a specific bioreceptor. Based on the silane–oxide chemistry, the process is transferable to any kind of surface that can be either oxidized in surface or activated with O_2_-plasma, for instance. For this proof of concept, we have chosen to develop our biointerface on titanium and lithium niobate surfaces. The development of the biointerface consists of grafting antibodies via a self-assembled monolayer (SAM) composed of an aminopropyltriethoxysilane (APTES) and a linker (phenylene diisothiocyanate, PDITC). Two functionalization routes were tested for grafting APTES: in anhydrous toluene followed by a heating step at 110 °C or in chloroform at room temperature. The results obtained on titanium show comparable grafting efficiency between these two routes, allowing us to consider the transposition of the route at room temperature on lithium niobate. The latest route was chosen for fragile materials that do not require the heating steps necessary when using toluene for grafting aminopropyltriethoxysilane. Different surface characterization techniques were used, such as IR spectroscopy (FTIR-ATR), X-ray photoelectron spectroscopy (XPS), and contact angle (WCA), to verify the successful grafting of each layer. Biodetection experiments in static conditions were also carried out to demonstrate the specificity of pathogenic detection, testing an ideal medium with solely bacteria, with no other food sampling nutrients. This paper demonstrates the successful elaboration of a biointerface using APTES as the first anchoring layer, with chloroform as a mild solvent. The process is easily transferable to any kind of fragile surface. Moreover, following anti-*L. monocytogenes* antibodies, our biointerface shows a specificity of capture in static mode (at a concentration of 10^7^ CFU/mL for an incubation time of 4 h at 37 °C) of up to 98% compared to a species negative control (*E. coli*) and up to 85% in terms of strain specificity (*L. innocua*).

## 1. Introduction

Food contamination by pathogenic agents is a global issue. According to the World Health Organization, there are 600 million cases of foodborne illness each year, resulting in 420,000 deaths [1]. Detecting these pathogenic bacteria has, thus, become a major challenge for the food and health industries [2].

Conventional methods to monitor microbial contamination are culture-based, which are time-consuming (24 h to 72 h) [3], costly, and require a large number of samples [4,5]. Researchers have developed many alternative methods: Enzyme-Linked ImmunoSorbent Assay (ELISA) [6], Polymerase Chain Reaction (PCR) [7], and Lateral Flow Immunoassay (LFIA) [8], among others [4,9]. These methods face several limitations, including sensitivity, cost, and, notably, measurement time. As a result, it is difficult to meet the demand for large-scale bacterial detection in food samples with current technologies.

New technologies have been developed for more sensitive, specific detection and to reduce the measurement time. The multiplex detection capability is included as an important property of the detection technologies. Among them, biosensing devices are developed with their applications in tackling the top challenges in food production and sustainability [10]. A biosensor is an analytical device used to quantify molecules of interest in a biofluid. It comprises a bio-recognition element (aptamer, enzyme, antibody, etc.), which is specific to the target. A transducer converts the physiological or biological signal due to the recognition of the target with the ligand into a physical (colorimetry, fluorescence, SPR, etc.) or electrical (impedance, capacitance, etc.) signal [11]. The performance of the biosensor depends on both the quality of the transducer and that of the biorecognition surface.

In this study, we propose to develop an acoustic wave biosensor [12]. This type of transduction is highly valued for its sensitivity, robustness, and reliability [13]. Currently, quartz is the most commonly used material for acoustic transduction. There are several types of acoustic wave sensors, but the most popular one for detecting biologically relevant elements is the Quartz Crystal Microbalance (QCM). In our team, we have developed biosensors using other piezoelectric materials for transduction: ZnO, GaAs, and LiNbO_3_ (LN); these materials have higher piezoelectric coefficients, which suggests the potential for higher sensitivity values [14,15]. In this study, we focus on the LN-based QCM.

The purpose of this article is to demonstrate how we elaborated and optimized a specific biointerface (surface functionalization and bioreceptor immobilization) on LN. This crystalline material is pyroelectric, which means that if the temperature of the material rises during the biointerface fabrication process, stresses will be generated that may damage the transducer. To avoid any degradation of the substrate, we, therefore, have to establish the biointerface using a room-temperature process.

Various surface modification strategies are available; among them, covalent immobilization has great potential. The covalent immobilization method has many advantages compared to others, such as physical way (adsorption, electrostatic, Van der Waals, etc.). Since LN is an oxide, we propose to develop a chemically grafted biointerface based on organosilanes. The silanization process consists of four steps. They are hydrolysis, condensation, hydrogen bond formation, and drying/curing. The silanization process with 3 aminopropyltriethoxysilane (APTES) on oxide surfaces is the most used [16,17,18], often carried out using anhydrous toluene as solvent, with the curing step being a heating process up to 110–120 °C. Optimizing the deposition process of the APTES layer to have a well-oriented monolayer is crucial to obtain a stable surface and to have the opportunity to chemically attach the bioreceptor to the silane layer via a linker. Moreover, a thick layer of APTES induces a fragile structure and could impact the sensor performance, especially for an acoustic wave sensor, where the depth penetration is a critical parameter [19]. The ideal condition for obtaining monolayers of APTES on LN is still not clear and we have to determine the best silanization process for our application, with a recipe that avoid any curing step by heating process as LN is pyroelectric; Indeed, it can degrade itself upon fast temperature variation and/or heating above 70–80 °C, making thus the toluene route not suitable for LN. Several processes are used for APTES monolayer formation. The deposition method, concentration of APTES, type of solvent used for the solid phase deposition, deposition time, temperature, pH, and drying conditions have a great influence on the properties of the monolayer as explained by Issa and Luyt [20].

The main purpose of this paper is to demonstrate the potential of a generic antibody-based biointerface elaborated in mild conditions suitable for fragile substrates such as LN and to establish a proof of concept towards the specific capture of *Listeria monocytogenes*, a highly virulent bacterium for humans.

**Figure 1:**
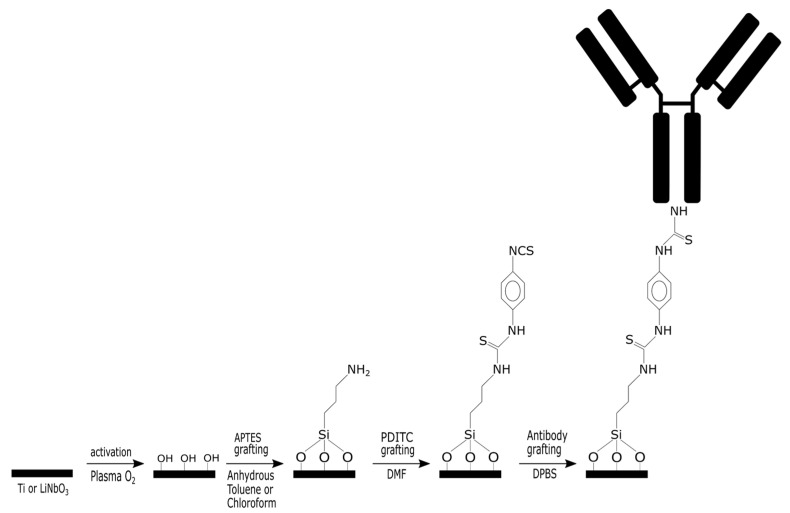
Overall grafting strategy of antibodies on APTES self-assembled monolayers via a diisocyanate linker.

Given the relatively high cost of the LN, we chose to conduct the initial tests on a titanium sample, which is naturally covered by a native oxide layer. The methodology used is as follows and is detailed in Figure 1:

(i) Reproduce a biofunctionalization process of the native titanium oxide using a protocol that employs anhydrous toluene as a solvent during the deposition of APTES, followed by a temperature increase. PDITC [21] is used as a linker for the immobilization of the polyclonal antibody targeting *Listeria monocytogenes*.

(ii) Develop a biofunctionalization process of the native titanium oxide using a protocol that employs chloroform as a solvent for APTES deposition at room temperature with no need for a heating step. The grafting steps for PDITC and the antibody remain identical to the previous case.

A characterization of the surface after each step of the biointerface elaboration up to antibody immobilization will be conducted to validate the second protocol on titanium.

(iii) Adapt the room-temperature chloroform-based protocol to the surface of LN.

A comparison of the characterization results after each step will allow the validation of the protocol on our piezoelectric material.

In this study, the experimental developments will be carried out up to the capture of *Listeria monocytogenes* (*L. monocytogenes*). The physicochemical characterization tools used for the systematic analysis of surfaces after each step are: water contact angle measurement (WCA), X-ray photoelectron spectroscopy (XPS), and Fourier-transform infrared spectroscopy (FT-IR). The capture of bacteria is confirmed by optical microscopy after incubation with the bacterial inoculum. This proof of concept will be conducted using ideal media containing only bacteria in PBS.

## 2. Results and Discussion

### 2.1. Biointerface Elaboration on Titanium Surfaces

The first part of our study focused on comparing the formation of an APTES self-assembled monolayer using two different strategies: a classical one using anhydrous toluene followed by a heating step versus a chloroform route at room temperature.

**Figure 2:**
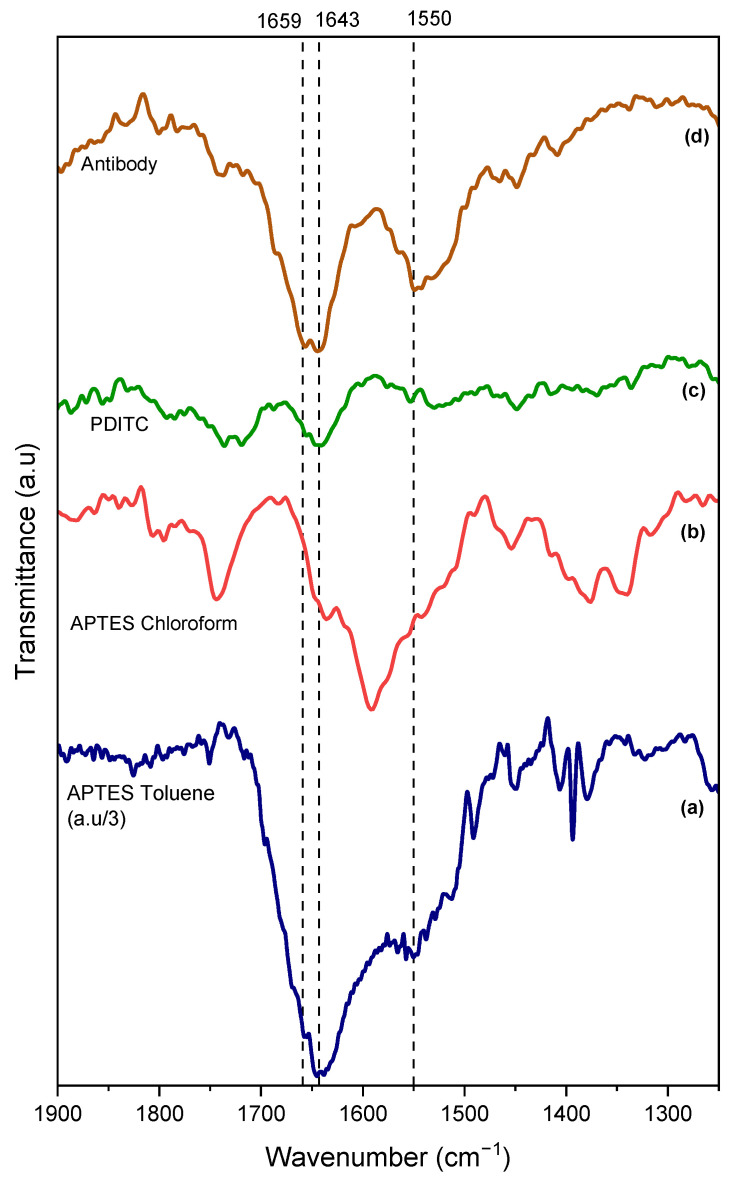
FT IR-ATR spectra of APTES-titanium functionalization via the toluene route (**a**) and the chloroform route (**b**). (**c**,**d**) PDITC and antibody steps following the APTES-chloroform route.

We followed the different steps of grafting by infrared spectroscopy, as shown in Figure 2.

As can be seen on the APTES spectrum, we find the characteristic bands expected for an amino-silane monolayer, in particular a broad main peak in the 1500–1700 cm^−1^ region, assigned to the δΝH_2_ vibration but also protonated amine groups—symmetric and asymmetric deformation [22]. The FT-IR data are confirmed by the change in wettability of the surface following the adsorption of APTES. Indeed, as can be seen in Table 1, the contact angle measurements on Ti-APTES are 43 ± 12°, which is consistent with values reported in the literature. The first step of surface functionalization by a monolayer of APTES is therefore confirmed.

With the aim of creating biointerfaces using “milder conditions”, we relied on the literature that offers alternatives to aggressive organic solvents and harsh (anhydrous) conditions, but which also avoid a temperature increase step that can be harmful to certain piezoelectric materials.

Thus, we adapted a functionalization strategy for APTES using chloroform as the solvent; this strategy offers notable advantages: the solvent is not anhydrous, and it does not require a temperature-controlled drying/crosslinking step. It is also important to note the addition of a chemical (TCA) that prevents silane hydrolysis, such as the role of anhydrous toluene. The IR spectrum presented in Figure 2 for this APTES grafting route in chloroform exhibits the same characteristic peaks, particularly the broad feature in the 1500–1700 cm^−1^ region, as expected. Additionally, the water contact angle of 46 ± 6° (Table 1) matches that obtained using the anhydrous toluene method, confirming that chloroform enables the formation of an equivalent self-assembled monolayer. It is important to note the difference in intensity between the spectra of APTES depending on the solvent. In fact, intensities observed for toluene (spectrum divided by three in Figure 2 for better clarity) solvent are much higher than those obtained with chloroform, suggesting formation of multilayers of APTES [20,23]. Therefore, for better clarity, the following spectra (PDITC and Antibodies) correspond to the samples prepared using APTES grafting carried out in chloroform.

The next step consists of grafting a linker from the diisocyanate family with a (rigid) aromatic ring at its center to avoid any bridging.

The FT-IR spectrum obtained after the PDITC functionalization step shows clear changes compared to the previous step, notably the presence of a main feature at 1643 cm^−1^. This band potentially originates from the reaction between the amine groups (protonated or not) of APTES and the PDITC to form a chemical bond similar to an amide or urea bond, in this case –NH-C=S-NH-. This suggests that PDITC has successfully reacted on the surface. Moreover, contact angle measurements tend to confirm this trend, showing an increase to 87 ± 4° for the chloroform-based route vs. 70 ± 10° for the toluene-based method. These values are consistent with those reported in the literature.

The final step in the development of the antibody-based biointerface also reveals a strong evolution of the infrared spectrum, characterized by the appearance of two peaks at 1659 cm^−1^ and 1550 cm^−1^ corresponding to the amide I and amide II bands, respectively. These features are characteristic of the amide I and amide II bonds present in the antibody structure. An evolution of the contact angle measurement also confirms the presence of antibodies on the titanium surface with an expected decrease from approximately 70–90° to approximately 60–70° ± 5–6°. All these results confirm the successful development of an antibody-based biointerface on an APTES-SAM in a classic organic solvent such as anhydrous toluene.

**Table 1:** WCA values (in degrees) at the different steps of functionalization for Ti and LN.

	Rough Surface	APTES		PDITC		Antibodies	
	measured	measured	expected	measured	expected	measured	expected
Ti (Toluene)	29 ± 6	43 ± 12	47–70 [24,25,26]	70 ± 10	60–84 [24,27]	60 ± 5 (*E. coli*)	56–69 [28,29]
Ti (Chloroform)	20 ± 3	49 ± 6		87 ± 4		71 ± 6 (*E. coli*)	
LN (chloroform)	42 ± 2	40 ± 5		46 ± 6		59 ± 9 (*E. coli*)	

To confirm the successful formation of the APTES-PDITC-antibody biointerface, both using anhydrous toluene and chloroform for the first APTES step, XPS experiments were carried out on each of the three stages for both solvent routes.

**Figure 3:**
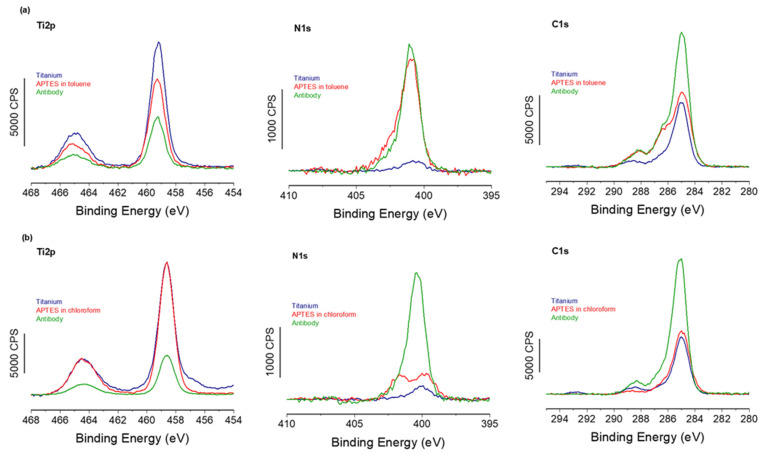
XPS results for the three grafting steps on titanium surfaces: (**a**) toluene route, (**b**) chloroform route, showing high-resolution spectra for Ti2p, N1s, and C1s regions.

Starting first with the toluene route, Figure 3 shows a strong decrease in the Ti2p signal with a corresponding increase in the N1s signal between the different functionalization steps for both toluene and chloroform solvents. This confirms, firstly, the growth of the organic layer deposited on the titanium surface. Secondly, by examining the elemental composition of this organic layer (Table 2) and comparing the experimental and theoretical % values, we can clearly see that the experimental composition is close to that expected for an ideal layer, confirming again the successful grafting of the antibodies. In addition, the C1s high-resolution region shows a feature at high binding energy, ~ 288 eV, usually assigned to the C=O of the amide bond within antibodies [30]. XPS provides, among other functions, the ability to quantify the surface density of the grafted elements. Thus, through Equation 1, we were able to evaluate the antibody recovery rate on the biointerface developed using the classic anhydrous toluene route, with a recovery rate of 85%, which is equivalent to approximately 3.8 × 10^3^ antibodies/µm^2^; see Table 3.

Furthermore, a comparison of the XPS spectra from samples with APTES grafted using toluene and chloroform (Figure 3) reveals that the spectra are almost identical, strongly suggesting identical organic layers grafted on top of the silane SAMs. In addition, XPS-based calculations of the antibody coverage rate give a value very close to 86% and an identical antibody density of 3.8 × 10^3^ antibodies/µm^2^, as shown in Table 3. These values are consistent with maximal surface coverage of antibodies estimated by several surface analysis techniques (ellipsometry, TOF-SIMs, etc.) and ranging from 10^4^ to 10^5^ antibodies/µm^2^ [31].

These results confirm that both toluene and chloroform routes lead to the same final biointerface composed of antibodies grafted at a similar coverage rate.

### 2.2. Biointerface Elaboration on Lithium Niobate Surfaces

The chloroform-based strategy was, therefore, applied to LN, a piezoelectric and pyroelectric material, which would not withstand temperature increases as required in the anhydrous toluene route. The wettability results obtained by the WCA measurements (Table 1) show values that vary depending on the functionalization step. More importantly, these values are very close to those obtained on titanium and consistent with those reported in the literature.

**Figure 4:**
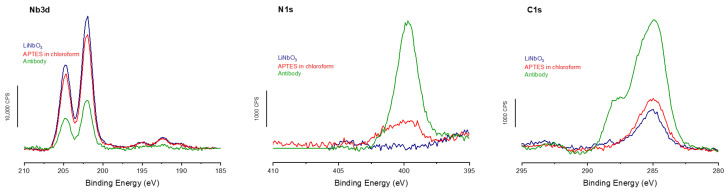
XPS measurements of the three grafting steps on LN surfaces showing high-resolution spectra for Ti2p, N1s, and C1s regions.

The XPS analyses performed at the different grafting stages (Figure 4) show the same trends as those observed on titanium. Specifically, a decrease in the signal from the LN substrate is accompanied by an increase in both N1s and C1s signals. These results confirm the successful grafting at each of the three stages of the biointerface formation, with a decrease in the niobium signal and an increase in the nitrogen signal at 400 eV. In addition, the carbon signal rises above the expected characteristic peak at 288 eV, corresponding to the contribution of the antibody amide bonds.

Finally, the XPS calculations show a lower antibody coverage rate in the case of LN, with only 76%, but a surface antibody density relatively close to the two previous surfaces, with 3.3 × 10^3^ antibodies/µm^2^, which remains close to the expected maximum value of 4.4 × 10^3^ antibodies/µm^2^ (assuming the shape of an antibody to be a perfect cube with an edge of 15 nm [32] and without taking into account possible variation in the adsorption geometry of our given antibodies) on these surfaces functionalized with APTES.

These experiments made it possible to implement an APTES grafting strategy on titanium surfaces using chloroform as a solvent that does not require heating. This is used with the addition of TCA to prevent hydrolysis of silane bonds, which does not occur with anhydrous solvents. The grafted antibody densities were found to be similar for the two types of solvents used for the formation of the APTES SAM, allowing us to conclude on identical biointerfaces. This gentler approach was therefore used to functionalize samples that are more fragile than titanium, and especially do not support a heating step. Thus, the antibody-based biointerface was also successfully developed on LN, resulting in a monolayer of antibodies with a density approximately 10% lower than that observed on titanium.

LN is used with the aim of developing dynamic pathogen sensors based on acoustic sensing technologies. However, a validation step for the specific capture of pathogens under dynamic and controlled conditions must first be established, as presented below.

### 2.3. Microbiological Detection Assays 

The antibodies used in our biointerface are polyclonal antibodies targeting *Listeria* species, and more specifically, the *monocytogenes* strain, a Gram-positive bacterium. To test the efficiency of our biointerface, we exposed it to an overnight-grown inoculum of *Listeria monocytogenes* (10^7^ CFU/mL at 37 °C). To assess its specificity, we also exposed it to *L. innocua* to determine the strain specificity. Finally, to determine species specificity, we inoculated our surfaces with *E. coli*, a Gram-negative bacterium.

**Figure 5:**
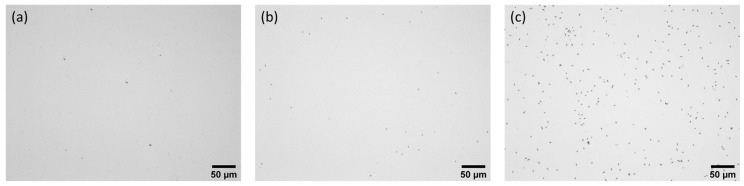
Optical microscopy images of LN surfaces functionalised with anti-*L. monocytogenes* antibodies inoculated with (**a**) *E. coli* (species specificity), (**b**) *L. innocua* (strain specificity), and (**c**) *L. monocytogenes* (specific bacterium).

The results were obtained by counting bacteria using optical microscopy after staining for better contrast, as can be seen in Figure 5, where each rod represents a bacterium. Qualitatively, we can note the very low quantity of *E. coli* (left) compared to the two images on the right (Listeria). Similarly, *L. monocytogenes* appears to have more bacteria than *L. innocua*.

**Figure 6:**
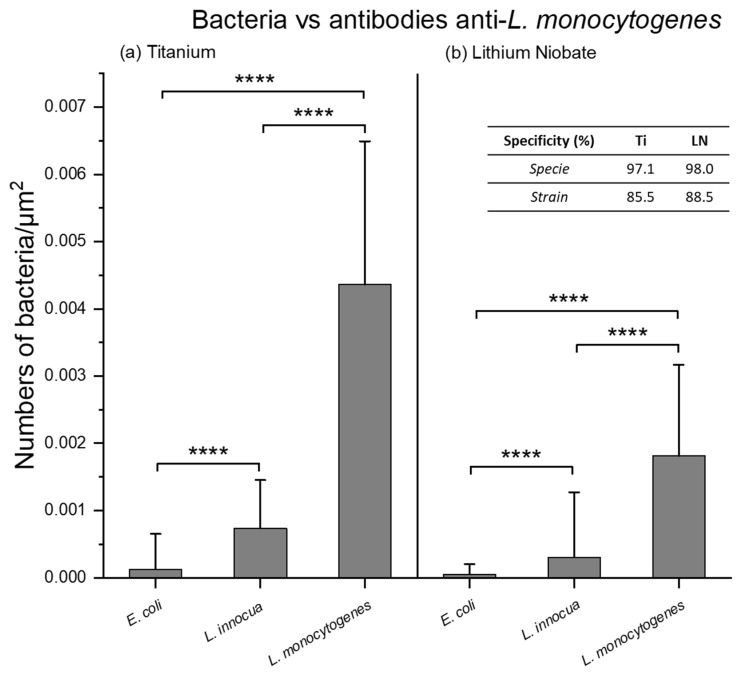
Statistical analyses of the number of bacteria counted per area unit on titanium (**a**) and LN (**b**) for *E. coli* (species specificity), *L. innocua* (strain specificity), and *L. monocytogenes* (specific bacterium). For each condition, the data presented the average count of bacteria on 270 optical microscopy images, explaining the high error bars visible on the graphic. (****: *p* < 0.001).

A statistical analysis was therefore carried out on around thirty randomly selected images from all the analyzed surfaces. The results, expressed in number of bacteria/µm^2^, are presented in a graph for the titanium and LN surfaces functionalized with anti-*L. monocytogenes* antibodies (Figure 6).

These quantitative analyses reveal the same trend as that observed in the microscopy images, with very few *E. coli* bacteria captured in comparison to the two *Listeria* strains. These trends are identical for the two tested surfaces, titanium and LN, confirming once again the strong performance of the biointerfaces on two completely different oxide surfaces. Focusing now on the specificity of these biointerfaces, it is possible to quantify capture specificity relative to a defined control using the following Equation (1):

(1) $${\%\;Specificity}_{A-B}=\;\left[1-\left(\frac{number\;A}{number\;A+number\;B}\right)\right]\;\times 100$$

where *A* and *B* represent two different bacteria, *number A* represents the amount of bacteria *A* and *number B* represents the amount of bacteria *B*.

This approach first enables the evaluation of the specificity of anti-*L. monocytogenes* antibodies for the capture of *L. monocytogenes* (Gram-positive bacterium) compared to a Gram-negative species, *E. coli*. The resulting specie specificity values are 97.1% for titanium and 98.0% for LN surfaces, respectively.

These high specificity values nevertheless remain a textbook case because the two bacterial species tested are very different from each other. We, therefore, also considered it interesting to examine this specificity between two bacterial strains of the same species: *Listeria innocua* vs. *Listeria monocytogenes*, because it is known that cross-recognition is possible between strains, even for an antibody, although polyclonal, but clearly targeting a single strain of a given species. Thus, the strain specificity of a biointerface consisting of anti-*L. monocytogenes* antibodies immobilized on titanium and LN surfaces are 85.5% and 88.5%, respectively.

If a cross-comparison is made between the two titanium and LN surfaces functionalized by the “soft” route using chloroform for the first step, very similar detection efficiencies were obtained with, respectively, 1.14 × 10^−7^ vs. 0.85 × 10^−7^ bacteria/antibody for Ti and LN. Again, assuming that a single bacterium occupies approximately 0.4 µm^2^ versus the area covered by an Ab (~200 nm^2^), the ratio bacterium/Ab arises to five orders of magnitude. In other words, this implies that only 1 in every ~1000 grafted antibodies binds to a bacterium, suggesting that a possible reduction in antibody coverage could potentially be achieved for an equivalent detection efficiency, assuming again the same affinity for the diluted surface density of antibodies.

Finally, we can also note that the LN surface exhibits higher specificity, despite having a lower antibody density and a lower detection efficiency. This could be attributed to a better organization of the antibodies on the oxide surface resulting from the more crystalline structure of LN compared to native titanium oxide. The crystalline structure may promote a better orientation of the antibodies, enhancing their reactivity during interactions with bacteria.

## 3. Materials and Methods

### 3.1. Chemical

Acetone, ethanol, and anhydrous toluene were purchased from VWR (*p*-phenylene diisothiocyanate (PDITC) and chloroform from Fisher Chemical (Illkirch, France); 3-aminopropyltriethoxysilane (APTES) from TCI chemicals (Paris, France); trichloroacetic acid (TCA), anhydrous *N*,*N*-dimethylformamide (DMF), anhydrous pyridine, and Dulbecco’s Phosphate-Buffered Saline (DPBS) from Sigma-Aldrich (Saint-Quentin-Fallavier, France); and anti-E. coli rabbit polyclonal antibodies and anti-*Listeria monocytogenes* rabbit polyclonal antibodies from Abcam (Cambridge, UK). All the reagents were used without further purification, and the experiments were carried out at room temperature unless otherwise specified.

Titanium substrates (10 mm × 10 mm) were mirror-polished, and LN substrates were purchased from Roditi (London, UK) and cut to 10 mm × 12 mm in the Mimento RENATECH Technology Center cleanroom.

### 3.2. Sample Preparation 

Before any surface functionalization, the titanium (Ti) and LN substrates were sonicated in acetone (2 × 5 min), dried under a nitrogen flow, sonicated in ethanol (2 × 5 min), and then dried again under a dry nitrogen flow.

The cleaned surfaces were first activated using O_2_ plasma (PVA TePla, Wettenberg, Germany) for 10 min, and then either immersed overnight in a solution containing 2% APTES and 0.28 g TCA in HPLC-grade chloroform under magnetic stirring. The surfaces were rinsed by sonication (VWR, Fontenay-sous-Bois, France) in HPLC-grade chloroform (2 × 5 min) in a 50:50 mixture of Milli-Q water and HPLC chloroform (2 × 5 min), and finally dried under a dry nitrogen flow or immersed in a solution containing 2% APTES in anhydrous toluene. The surfaces were then rinsed under stirring in anhydrous toluene (2 × 5 min) and dried under a dry nitrogen flow. After the rinsing, the samples were heated at 110 °C for 20 min.

The amine-terminated substrates were functionalized via droplet deposition (100 µL) with a mixture of PDITC (3 mg), anhydrous pyridine (150 µL), and anhydrous DMF (1.35 mL) for 1 h. They were then rinsed by sonication in anhydrous DMF (3 min), followed by absolute ethanol (2 × 5 min), and finally dried under a dry nitrogen flow.

Antibody immobilization was performed by droplet deposition (100 µL) of the antibody solution (50 mg/L in DPBS) onto the modified Ti and LN substrates for 2 h. The surfaces were then rinsed by sonication in DPBS (3 min), rinsed under stirring in absolute ethanol (2 × 5 min), dried under a dry nitrogen flow, and stored at 4 °C.

### 3.3. Microbiological Tests and Bacterial Strains

Agar powder was purchased from Biokar Diagnostics (Beauvais, France), Difco LB broth from BD (Le Pont-de-Claix, France), and Brain Heart Infusion (BHI) broth from Sigma-Aldrich (Saint-Quentin-Fallavier, France).

The bacterial strains used in this study were *Escherichia coli* (*E. coli*) ATCC 25922, *Listeria innocua* (*L. innocua*), and *Listeria monocytogenes* (*L. monocytogenes*), both isolated from milk matrices. The strains were stored at −80 °C in glycerol aliquots. The inoculum was prepared by growing colonies on agar media: LB agar (15 g/L agar + 25 g/L LB) for *E. coli* and BHI agar (15 g/L agar + 37 g/L BHI) for both *Listeria* strains. Petri dishes were incubated overnight at 30 °C for *E. coli* and at 37 °C for both *Listeria* strains.

Liquid cultures were initiated by transferring one colony from the solid medium to 10 mL of the corresponding liquid medium (LB at 25 g/L, BHI at 37 g/L), and then incubated overnight at 30 °C (*E. coli*) or 37 °C (*L. innocua* and *L. monocytogenes*) under agitation at 90 rpm.

Bacterial concentration after overnight culture or after centrifugation and harvesting was carefully evaluated using UV-Vis measurements (Shimatzu Corporation, Kyoto, Japan) of Optical Density (OD) at 620 nm and by spreading these inocula on an agar plate followed by numeration.

### 3.4. Static Contact Detection

The Ti and LN samples were washed with 70% ethanol and dried in a sterile environment.

Exponentially growing bacteria were harvested by centrifugation (Eppendorff, Hambourg, Germany) (5000× *g*, 5 min, 25 °C), washed twice with sterile PBS, and resuspended in PBS to a concentration of 10^9^ CFU/mL. A working solution was prepared for each strain at 10^7^ CFU/mL in PBS. Each sample was immersed in 4 mL of the bacterial suspension in a 12-well plate and incubated for 4 h at 30 °C (*E. coli*) or 37 °C (*L. innocua* and *L. monocytogenes*) under 90 rpm agitation.

Following incubation, the samples were washed three times with sterile PBS and dried under a gentle flow of dry nitrogen.

After incubation with the bacterial solutions, each sample was immersed for 10 min in a homemade 0.5% crystal violet solution in ethanol. After staining, the surfaces were rinsed with milli-Q water until the color was no longer visible in the well water, and then the surfaces were dried with dry nitrogen.

### 3.5. Characterization Techniques

#### 3.5.1. ATR-FTIR

For FT-IR spectroscopy characterization, a Diamond ATR modulus from Perkin-Elmer SpectrumTwo was coupled to an FT-IR instrument (Perkin Elmer, Waltham, MA, USA). The titanium samples were placed on top of the ATR crystal and pressed for intimate contact between both surfaces; thus, the reflected light was focused on a DTGS (Deuterated TriGlycine Sulfate) wide band detector. Spectra were acquired at a resolution of 8 cm^−1^ by a co-addition of 60 spectra (1 min acquisition time). After making the ratio between the sample spectrum and a background collected in air, a basic baseline correction was applied to flatten the spectra; no further smoothing corrections were applied. The spectra were plotted as % transmission.

#### 3.5.2. XPS Analyses

The XPS analyses for the titanium samples were performed using a Scienta Omicron (Uppsala, Sweden) Argus X-ray photoelectron spectrometer equipped with a monochromated AlKα radiation source (hν = 1486.6 eV), and 150 W electron beam power. The emission of photoelectrons from the sample was analyzed at a takeoff angle of 45° for Omicron Argus X-ray under ultra-high vacuum conditions (≤10^−9^ mbar). Spectra acquisition was carried out with 100 eV of pass energy for the survey scan and 20 eV of pass energy for the C1s, O1s, N1s, Si2p, S2p, and Ti2p regions.

The XPS analyses for the LN samples were performed using a Thermo Fisher (Thermo Fisher Scientific, Waltham, MA, USA) Alpha 110 photoelectron spectrometer, equipped with a standard AlKα non-monochromated source, operated at a 300 W electron beam power. The emission of photoelectrons from the sample was analyzed at a takeoff angle of 90° under ultra-high vacuum conditions of ≤10^−9^ mbar. Spectra acquisition was carried out with 100 eV pass energy for the survey scan and 20 eV pass energy for the C1s, O1s, N1s, Si2p, S2p, and Nb3d.

Binding energies (BEs) were calibrated against C 1s aliphatic carbon bonds (C–C and CH) binding energy at 284.8 eV, and element peak intensities were corrected according to Scofield factors [33]. The spectra were fitted using the Casa XPS v.2.3.15 software (Casa Software Ltd., Cardiff, UK) and applying a Gaussian/Lorentzian ratio equal to 70/30. The peak areas were determined after subtraction of a Shirley background.

The evaluation of antibody coverage (*θ*) was calculated using the following equation:

(2) $$\frac{I_{N1s}}{I_{S}}\;=\;\;\frac{1320\;\frac{\rho_{Ab}}{M_{Ab}}\;\sigma_{N1s}\lambda_{N1s}^{Ab}\;\theta \left[1-exp\left(-\;\frac{d_{Ab}}{\lambda_{S}^{Ab}}\right)\right]}{\frac{\rho_{S}}{M_{S}}\;\sigma_{S}\;\lambda_{S}^{S}\;\left[\theta \;exp\left(-\;\frac{d_{Ab}}{\lambda_{S}^{Ab}}\right)+\left(1-\theta \right)\right]\;exp\left(-\;\frac{d_{org}}{\lambda_{S}^{org}}\right)}$$

where *I_x_* is the raw intensity of element *x*. *ρ_Ab_* and *ρ_s_* are the densities of the antibody and substrate, respectively. *d_Ab_* and *d_org_* are the thicknesses of the antibodies and the full organic layer, respectively. The Scofiel photoionization cross-sections *σ* are equal to 1.8 for N1s, 7.021 for Ti2p, and 8.21 for Nb3d [33]. *M_Ab_* and *Ms* are the molecular weights of the antibody and the substrate, respectively, and finally, $\lambda_{x}^{y}$ is the inelastic mean free path of electrons *x* in the matrix *y*. These were calculated using the Quases program (QUASES-IMFP-TPP2M Ver.3.0, Sven Tougaard, Odense, Denmark) based on the TPP2M formula.

#### 3.5.3. Optical Microscopy

Bacterial detection was assessed by analyzing the Ti and LN surfaces that had undergone contact tests. Each surface was observed using a Nikon ECLIPSE LV100ND optical microscope (Nikon Corp., Tokyo, Japan) at magnifications of ×50 and ×100. Ten photos were taken at each magnification for each sample of LN, and thirty photos at ×100 magnification for each sample of Ti. Bacterial counts per image were conducted using the Cell Counter plugin in the ImageJ software (NIH, Ver. IJ1.46r, Bethesda, MD, USA). Each count was then divided by the surface area of the corresponding image. Based on these results, the mean value, standard deviation, and statistical tests (Fisher’s and Student’s *t*-tests) were calculated for each bacterial strain.

## 4. Conclusions

In this study, we have developed specific antibody biointerfaces based on APTES monolayers using two very distinct organic solvents for the first step of APTES functionalization. On titanium surfaces, we have demonstrated that both routes successfully allow the grafting of the same amount of antibodies. For transposition to LN, a pyroelectric material, we have chosen the less aggressive solvent (chloroform), which does not need a heating step. Once again, we have shown the successful elaboration of an antibody biointerface on LN surfaces, achieving a comparable surface coverage of antibodies to that on titanium substrates. This opens great opportunities for surface chemistry and biointerface elaboration on very fragile and expensive oxide materials such as LN.

In addition, we have demonstrated that the antibody biointerfaces were capable of performing static biodetection upon pathogenic bacteria with a very high specificity rate: up to 97% in terms of species (*E. coli* vs. *L. monocytogenes*) and also a very good strain specificity up to 88% between the targeted bacterium (*L. monocytogenes*) vs. an opportunistic strain from the same species (*L. innocua*).

The good results obtained in this study open the door to the development of pathogenic bacteria biosensing devices, such as QCM based on LN. Future work will focus on the dynamic capture of bacteria on LN surfaces using the "soft" biointerface developed with chloroform, in order to enable real-time, flow-based monitoring of potential fluid contamination, particularly for applications in the food industry. However, when looking at the potential industrialization process of such biosensors, several issues have to be considered, such as (i) the cost of the sensor and its manufacturing process, (ii) the lack of robustness and reliability of the biointerface during immunoassays. To reduce the cost of the sensor, we are considering regenerating the biointerface chemically so that it can be reused for several successive tests. Preliminary regeneration experiments have already been conducted. To improve the robustness of the biosensor when analyzing a complex fluid, we are currently developing a biosensor with multiple different biointerfaces simultaneously exposed to our sample. This will allow us to multiplex the measurements, on the one hand, by replicating identical measurements, and on the other hand, by testing for the presence of other targeted pathogen bacteria. Analyzing the extensive data generated through multiplexing will enable us to obtain a reliable and robust outcome.

## Figures and Tables

**Table 2:** Elementary atomic percentages (%) obtained from XPS data for all three functionalization steps on titanium surfaces (two APTES routes) and LN.

Theoretical Atomic %	C1s	O1s	N1s	Si2p	S2p	--
APTES	16.7	50.0	16.7	16.6	--	--
Antibody	65.1	16.6	18.3	traces	traces	--
**Atomic % LN (chloroform)**	**C1s**	**O1s**	**N1s**	**Si2p**	**S2p**	**Nb3d**
Raw surface	3.2	42.4	1.4	0.5	--	52.5
APTES (chloroform)	3.3	43.5	2.9	1.5	--	48.8
APTES (chloroform) without substrate contribution	5.6	89.5	2.3	2.6	--	--
Antibody	14.0	50.4	6.5	1.1	0.3	27.7
Antibody without substrate contribution	19.8	62.0	14.6	2.8	0.7	--
**Atomic % Ti (toluene)**	**C1s**	**O1s**	**N1s**	**Si2p**	**S2p**	**Ti2p**
Raw surface	46.0	39.8	0.8	1.6	0.2	11.6
APTES (toluene)	51.0	31.4	6.1	5.5	0.6	5.4
APTES (toluene) without substrate contribution	75.3	12.4	6.7	5.3	0.4	--
Antibody	67.1	20.1	5.5	4.2	0.3	2.8
Antibody without substrate contribution	58.9	23.0	8.4	9.0	0.8	--
**Atomic % Ti (chloroform)**	**C1s**	**O1s**	**N1s**	**Si2p**	**S2p**	**Ti2p**
Raw surface	36.9	47.9	0.7	--	--	14.5
APTES (chloroform)	38.3	40.8	2.2	7.3	--	11.4
APTES (chloroform) without substrate contribution	51.8	37.5	1.7	9.1	--	--
Antibody	70.9	19.6	4.8	1.5	0.2	3.0
Antibody without substrate contribution	82.5	9.8	5.8	1.5	0.4	--

**Table 3:** Surface coverage and antibody density for all three strategies, deduced from XPS data.

	Ti (Toluene)	Ti (Chloroform)	LN (Chloroform)
Surface coverage (*θ*)	85%	86%	76%
Antibodies/µm^2^	3.8 × 10^3^	3.8 × 10^3^	3.3 × 10^3^

## Data Availability

Data will be made available upon request.
